# Vestibular Well-Being Benefits of a Single Session of Functional Neurology Intervention on Saccadic Stimuli Dysfunction

**DOI:** 10.3390/healthcare13090989

**Published:** 2025-04-24

**Authors:** Guillermo Escribano-Colmena, Jorge Rey-Mota, Sara Hadid-Santiago, Álvaro Ramos-Garrido, José Francisco Tornero-Aguilera, Vicente Javier Clemente-Suárez

**Affiliations:** 1 Independent Researcher, 28660 Boadilla del Monte, Spain; escribanocolmenaguillermo@gmail.com (G.E.-C.); jreymota@gmail.com (J.R.-M.); 2 Faculty of Sports Sciences, Universidad Europea de Madrid, Tajo Street, s/n, 28670 Madrid, Spain; sarahadidsantiago@gmail.com (S.H.-S.); alvaroramosgarrido@hotmail.com (Á.R.-G.); josefrancisco.tornero@universidadeuropea.es (J.F.T.-A.); 3 Grupo de Investigación en Cultura, Educación y Sociedad, Universidad de la Costa, Barranquilla 080002, Colombia

**Keywords:** functional neurology, saccadic stimuli, neuromuscular function, vestibular, neuroplasticity, pain

## Abstract

**Background/Objectives:** This study aimed to analyze the psychophysiological effects of functional neurology intervention on dysfunction in vestibular saccadic stimuli, focusing on its impact on muscle performance, psychophysiological arousal, and pain perception. **Methods:** Seventy-five healthy volunteer participants were randomly divided into two groups: an experimental group that received functional neurology treatment and a control group that did not. Both groups underwent the same evaluations at four distinct time points. Key measurements included pressure pain threshold (PPT), hand strength, critical flicker fusion threshold (CFFT), blood oxygen saturation, heart rate, and the number of saccadic stimuli tolerated until dysfunction in an indicator muscle (anterior deltoid). The functional neurology intervention involved proprioceptive reflexes, trigger point desensitization, and systemic approaches to rectify neuromuscular dysfunctions. **Results:** The results showed that the functional neurology intervention significantly increased the number of saccadic stimuli tolerated, from 3.6 ± 3.3 to 26.1 ± 8.7, indicating an improvement in neuromuscular endurance. Additionally, PPT readings exhibited an upward trend from baseline to post-intervention, with the final reading averaging at 10.2 ± 5.3 kgf, and hand strength measurements showed a modest but significant increase post-intervention. Notably, CFFT and blood oxygen saturation levels remained relatively stable, suggesting that the intervention’s primary impact was on neuromuscular performance and pain perception rather than on cognitive arousal or systemic oxygenation. Heart rate data indicated a decrease post-intervention, implying potential improvements in autonomic nervous system function. In contrast, the control group did not present significant changes in any of the psychophysiological parameters evaluated. These findings underscore the potential of targeted functional neurology treatments to enhance physical performance and provide valuable therapeutic benefits for neuromuscular and cognitive dysfunctions. **Conclusions:** Functional neurology interventions can effectively improve muscle endurance, pain management, and overall neuromuscular health, highlighting its relevance as a therapeutic modality in sports performance optimization and rehabilitation contexts.

## 1. Introduction

Functional neurology distinguishes itself from traditional techniques by its targeted approach to harnessing the adaptive capacities of the nervous system through neuroplasticity-driven interventions. Unlike conventional medical practices that predominantly rely on pharmacological or surgical solutions, functional neurology emphasizes non-invasive strategies such as proprioceptive reflex stimulation, trigger point desensitization, and visual–motor coordination exercises [1,2]. This approach integrates comprehensive neurological evaluations with tailored therapeutic techniques to address specific dysfunctions within the nervous system. By directly targeting neural imbalances, functional neurology enables practitioners to stimulate the formation and strengthening of neural pathways, offering holistic benefits that extend beyond symptom relief to foster enhanced neuromuscular performance and overall well-being [3,4]. These interventions stand apart due to their focus on the interconnected nature of sensory, motor, and cognitive systems, aiming to optimize functional recovery in conditions ranging from vestibular dysfunctions to chronic pain and sports injuries [5,6]. As such, functional neurology provides an innovative therapeutic paradigm, leveraging the principles of neuroplasticity to achieve outcomes unattainable through traditional treatment models [7,8,9]

Central to functional neurology is the exploitation of neuroplasticity, enabling the nervous system’s adaptation and restructuring through specific sensory, motor, or cognitive stimuli [3]. By adopting targeted therapeutic interventions, practitioners aim to correct neurological imbalances and enhance neural functionality without the need for drugs or surgery [4]. The continuous quest for methods to boost performance, mitigate injury risks, and expedite recovery highlights the relevance of functional neurology principles. Furthermore, the focus on maintaining equilibrium, vestibular and visual function, and proprioception is deemed essential, as these factors are foundational to an athlete’s ability to sustain balance, coordinate movements, and perform complex sports maneuvers with precision and agility [5,9].

The influence of the visual system extends beyond balance and spatial orientation, playing a pivotal role in modulating various physiological responses, including heart rate, muscle strength, cortical arousal, and pain perception. The visual system’s impact on heart rate is mediated through its connections with the autonomic nervous system. In particular, saccadic eye movements, rapid, jerk-like movements made by the eye to shift the line of sight and update the visual image, can lead to changes in cardiovascular function due to the involvement of the vestibulo-autonomic pathways [10]. Additionally, research has shown that visual inputs, including those from saccadic movements, are integral to motor control, influencing muscle strength through their impact on attention and motor planning [11]. The concept of cortical arousal, or the state of being awake and responsive to stimuli, is also influenced by visual inputs. Saccadic movements have been shown to facilitate shifts in attention and cognitive processing, which can modulate the level of arousal and attention through projections to the reticular formation and other areas of the cortex [12]. Lastly, the visual system’s role in pain perception has been explored, with findings suggesting that activation of the superior colliculus during saccadic movements can modulate pain thresholds, offering novel approaches to pain management [6]. This intricate interplay between the visual system, including saccadic movements, and various physiological and perceptual functions underscores the system’s importance beyond its traditional role in balance and highlights the potential for therapeutic interventions targeting the visual system to have wide-ranging effects on health and performance.

In the realm of human sensory perception and motor coordination, the role of saccadic eye movements emerges as a pivotal factor in normal daily activities. These rapid, jerk-like movements of the eye are essential for updating the visual scene, facilitating attention shifts, and contributing to effective spatial navigation. It is estimated that humans perform thousands of saccadic movements daily, underscoring their importance in our visual and cognitive systems. The efficiency of these movements is closely tied to the overall functionality of the visual system. Dysfunction in saccadic performance can lead to significant impairments in vision, perception, and, by extension, daily functioning. Studies have demonstrated that anomalies in saccadic movements are not merely symptomatic of underlying neural or ocular issues but can directly contribute to a broader spectrum of cognitive and perceptual dysfunctions [6,10]. The intricate relationship between saccadic movements and cognitive processes highlights the necessity for a well-functioning visual system, not only for basic visual tasks but also for maintaining cognitive health and effective sensory integration. This interdependence further emphasizes the potential consequences of visual system impairments, which can range from minor inconveniences in daily life to more severe disruptions in cognitive and perceptual abilities.

Recent studies have highlighted the integral role of saccadic eye movements in neuromuscular performance and sensory–motor integration. For instance, the superior colliculus, a midbrain structure, is pivotal in coordinating saccadic movements and integrating sensory inputs to guide motor responses, thereby influencing both eye and head movements during tasks requiring visual attention [13]. Additionally, the supplementary eye field within the frontal cortex has been implicated in the planning and execution of saccades, contributing to the synchronization of eye movements with other motor activities [14]. Furthermore, sensory–motor adaptation mechanisms play a crucial role in maintaining the performance of goal-directed movements, with the saccadic system serving as a valuable model for studying these adaptation processes [15].

Building upon the established interconnections between the visual system, physiological responses, and perceptual experiences, the present study aims to analyze the psychophysiological effects of repeated saccadic visual stimuli until muscle failure is induced in an indicator muscle, and to assess the potential modulatory effects of a single session of functional neurology intervention.

Specifically, we hypothesize that repeated saccadic visual stimuli will induce measurable changes in muscle performance, psychophysiological arousal, and pain perception, and that a functional neurology intervention will mitigate these changes by enhancing neuromuscular function and pain thresholds.

## 2. Materials and Methods

### 2.1. Participants

This study was a randomized controlled trial designed to assess the impact of functional neurology interventions on neuromuscular and psychophysiological responses. We analyzed 75 volunteer participants who were randomly assigned to either an experimental group that received the intervention or a control group that did not.

The experimental group consisted of 45 participants (22 females and 23 males) with a mean age of 32.4 ± 8.3 years (range: 22 to 48 years), a mean height of 170.0 ± 6.0 cm, a mean weight of 70.3 ± 14.3 kg, and an average BMI of 24.2 ± 3.9. The control group included 30 participants (14 females and 16 males) with a mean age of 26.1 ± 3.3 years (range: 20 to 35 years), a mean height of 177.1 ± 6.9 cm, a mean weight of 79.9 ± 10.9 kg, and a BMI of 25.4 ± 2.4.

Participants were recruited through local advertisements and university research networks within a university setting, ensuring accessibility to healthy adult volunteers who met the eligibility criteria. Eligibility was strictly limited to individuals aged 18 to 50 years who were free from neurological disorders, chronic illnesses, or any conditions that could influence study outcomes. Exclusion criteria included the use of medications affecting the nervous or musculoskeletal systems, engagement in vigorous physical activity within 24 h prior to testing, alcohol consumption within 48 h before the study, smoking, recent injuries, or surgeries undergone within the past six months. To standardize conditions, all participants fasted for at least two hours before the evaluations. All assessments were conducted during a single session lasting approximately 45 min, with the four evaluation moments performed sequentially, allowing for a standardized 10 min recovery period between specific phases to maintain consistent physiological conditions.

The study was conducted in a controlled research facility with an ambient temperature of 22.4 ± 0.4 °C and a humidity level of 41.4 ± 2.4%. Ethical approval was obtained from the University’s Bioethics Committee under code 2024-510, and the study adhered to the principles outlined in the Declaration of Helsinki.

### 2.2. Procedure

Participants underwent assessments at four distinct time points to evaluate the effects of the functional neurology intervention on neuromuscular and psychophysiological performance:

1. Baseline measurement (basal): Initial assessments were conducted to establish a foundational understanding of each participant’s psychophysiological state and physical capabilities. This phase served as the control condition, offering crucial baseline data for later comparisons;
2. Post-indicator muscle failure (pre-intervention): Participants underwent a specific test designed to induce failure in an indicator muscle, the anterior deltoid, in response to repeated saccadic eye movement stimuli. The test evaluated the integrity of the myotatic reflex, which normally functions as a corrective mechanism to sustain muscle contraction. Dysfunction in sensory input processing, whether from visual, vestibular, or proprioceptive sources, can lead to the reflex’s failure, resulting in muscle fatigue. Electromyography (EMG) provided insight into motor unit recruitment, but it did not directly assess reflexive neuromuscular responses [8,16]. Measurements were recorded immediately following muscle failure to capture neuromuscular responses to acute stress before intervention;
3. Post-functional neurology treatment: Participants in the experimental group received a tailored functional neurology intervention aimed at correcting neuromuscular imbalances and dysfunctions. This intervention leveraged neuroplasticity to enhance neural and muscular function. Post-treatment measurements were conducted to assess immediate physiological changes attributable to the intervention;
4. Post-indicator muscle failure (post-intervention): The final assessment involved re-exposing participants to the saccadic eye movement stimuli test to induce failure in the anterior deltoid. The goal was to compare the muscle’s neurological response pre- and post-intervention, assessing improvements in endurance and overall neuromuscular health.

Each evaluation session lasted approximately 45 min, with a 10 min recovery period between assessment phases. The total duration of the experimental intervention was one session, aligning with the study’s title and scope.

### 2.3. Instruments and Study Variables

Standardized instruments and procedures were used to obtain study variables:

- Body mass measurement: Body mass was measured using a SECA 714 scale (SECA GmbH, Hamburg, Germany) with a precision of 100 g and a measurement range of 0.1–130 kg. The scale was placed on a flat, stable surface and calibrated to zero before each use. Participants were instructed to remove their shoes and heavy clothing, standing upright and distributing their weight evenly between both feet;
- Isometric hand-grip strength: Hand-grip strength was assessed using a TKK 5402 dynamometer (Takei Scientific Instruments Co., Ltd., Tokyo, Japan). Participants were seated with their shoulders flexed at 0 degrees, elbows flexed at 90 degrees, and forearms in a neutral position. The highest value obtained from two attempts with the dominant hand was recorded [17];
- Pressure pain threshold (PPT): PPT was measured using a non-electrical pressure algometer (FPK 60, Wagner Instruments Inc., Greenwich, CT, USA). The tip of the algometer was applied perpendicularly to the muscle tissue, increasing pressure at a constant rate of 1 kg/s. Participants signaled immediately upon perceiving pain. Measurements were taken at predefined anatomical points, such as the midpoint of the anterior trapezius muscle [18];
- Cortical arousal levels: Cortical arousal was evaluated using the critical flicker fusion threshold (CFFT), assessed with a Lafayette Instrument Flicker Fusion Control Unit (Model 12021, Lafayette Instrument Co., Lafayette, IN, USA). Increases in CFFT were interpreted as indicators of heightened cortical arousal and enhanced information processing capacity, whereas decreases suggested potential central nervous system fatigue [19];
- Blood oxygen saturation and heart rate: Blood oxygen saturation and heart rate were measured with a Beurer PO 30 pulse oximeter (Beurer GmbH, Ulm, Germany) to monitor cardiovascular responses before and after the intervention [20];
- Saccadic stimuli: To assess the number of tolerated saccadic stimuli before neuromuscular dysfunction, participants fixated on a finger placed 30 cm in front of their eyes and then shifted their gaze rapidly to another finger positioned at a 90° angle to either side. The number of tolerated saccadic movements before dysfunction of the indicator muscle (anterior deltoid) was recorded during evaluation moments 2 and 4 [7].

### 2.4. Functional Neurology Intervention

In our study, each participant received a bespoke functional neurology intervention based on the distinctive principles of NeuroReEvolution^®^ (http://nre-therapy.com/, Boadilla del Monte, Spain), a method not widely practiced due to its specialized nature. This personalized treatment began with a thorough clinical evaluation, including oral and visual assessments and functional neurology tests on joints and their associated muscles, to identify each participant’s specific neurological disorders and then conduct the specific neurological intervention based on blink reflex. A certified practitioner, holding a Level III certification in the Functional Neurology Manual Muscle Test from NeuroReEvolution^®^, applied this intricate knowledge to address the unique nervous system dysfunctions identified in each subject. This precision in diagnosis and treatment ensured a tailored and efficacious therapeutic approach [8,9,16].

The core of functional neurology therapy lies in its focus on proprioceptive reflexes, trigger point desensitization, and a holistic, systemic approach. Proprioceptive reflexes, which are automatic responses of the nervous system to physical stimuli such as muscle stretching or tendon pressure, play crucial roles in maintaining posture, balance, and movement. Our intervention aimed to rectify any dysfunctions in these reflexes that could contribute to pain or impair mobility. Trigger points—areas within muscle tissue that, when sensitized, can cause pain or spasms in other body parts—were meticulously identified and treated. By employing manual techniques and targeted stimuli, the therapy worked to desensitize these trigger points, thereby recalibrating the nervous system’s response to alleviate pain and enhance functional recovery. Embracing a systemic view, our approach treated the body as an interconnected whole, acknowledging that a problem in one area could influence distant regions through the network of reflexes. This strategy aimed to address not only the immediate area of dysfunction but also any related areas that might contribute to the patient’s overall neuromuscular health [7].

The functional neurology intervention was applied in a single session lasting approximately 20 min per participant and was conducted immediately following the pre-intervention assessment. The intervention included targeted techniques aimed at modulating visuo-vestibular and proprioceptive inputs to restore neuromuscular function [21]. Specifically, the treatment consisted of the following:

- Manual proprioceptive activation: Light tactility and pressure in trigger points techniques were applied to stimulate afferent pathways involved in neuromuscular control;
- Blink reflex stimulation: Participants were exposed to controlled blink reflex exercises to enhance oculomotor integration.

Objective assessment of dysfunctions was conducted prior to treatment, focusing on the presence of altered muscle response to saccadic stimuli, indicative of impaired sensorimotor processing. The intervention aimed to restore the myotatic reflex, which typically ensures corrective muscle contraction in response to a stimulus. In cases of dysfunction, the reflex was absent or inconsistent, suggesting compromised neuromuscular integration. While functional neurology is a developing field with limited clinical validation, this study aims to contribute reproducible data regarding its potential applications. Future research should further investigate its role in clinical populations, particularly in neurological rehabilitation and performance enhancement.

### 2.5. Statistical Analysis

For the statistical analysis of our study, IBM SPSS Statistics software version 21.1 was employed. Descriptive statistics were computed to present means and standard deviations (SDs) for all measured variables. The normality of the data distribution was assessed using the Kolmogorov–Smirnov test. Upon confirmation of normal distribution, parametric tests were applied. A General Linear Model (GLM) was utilized to determine the effects of the interventions, with subsequent post hoc comparisons being adjusted using Bonferroni correction to control for the risk of Type I error due to multiple comparisons. Additionally, we conducted an independent samples *t*-test to examine differences between the experimental and control groups. To further examine the effect of the intervention, a General Linear Model (GLM) was used, incorporating group, sex, age, pressure pain threshold (PPT), hand strength, and heart rate (HR) as independent variables. We considered findings with a *p*-value of 0.05 or lower as statistically significant, indicating a meaningful difference or effect. The level of significance was set at *p* ≤ 0.05, indicating that results achieving this threshold would be considered statistically significant.

## 3. Results

The functional neurology intervention significantly increased the number of saccadic stimuli tolerated until dysfunction in an indicator muscle (anterior deltoid) from 3.6 ± 3.3 to 26.1 ± 8.7 (F = 450.575, *p* < 0.000, ηp^2^ = 0.888), showcasing a profound improvement in neuromuscular endurance. Additionally, PPT readings, indicative of the pressure pain threshold, exhibited an upward trend from baseline to post-intervention, with the final reading averaging at 10.2 ± 5.3 kgf. Hand strength measurements also showed a modest but significant increase post-intervention, further underscoring the multifaceted benefits of the functional neurology approach. Notably, the critical flicker fusion threshold and blood oxygen saturation levels remained relatively stable, suggesting that the intervention’s primary impact was on neuromuscular performance and pain perception rather than on cognitive arousal or systemic oxygenation. The heart rate data indicated a decrease post-intervention, implying potential improvements in autonomic nervous system function (Table 1). Finally, the control group maintained the number of saccadic stimuli tolerated until dysfunction in an indicator muscle between evaluation moments 1 and 4 (2.8 ± 3.1 vs. 2.9 ± 3.0). In addition, the control group did not present as significant in any of the psychophysiological parameters evaluated in the four evaluation moments, but there were significant differences between the number of saccadic stimuli tolerated until dysfunction in an indicator muscle between the experimental and control group in the post-treatment evaluation (26.1 ± 8.7 vs. 2.9 ± 3.0, respectively; *p* = 0.000). Additional independent variables were tested within the General Linear Model (GLM) to explore potential moderators of intervention effectiveness. However, the results showed that none of the additional variables (group, sex, age, PPT, hand strength, HR) significantly influenced the main outcome measure (all *p* > 0.05). This suggests that the improvements observed were attributable to the functional neurology intervention itself, rather than being confounded by differences in participant characteristics.

## 4. Discussion

In this study, we set out to explore the psychophysiological effects of functional neurology intervention on saccadic stimuli dysfunction, specifically investigating its impact on an indicator muscle’s performance, psychophysiological arousal, and pain perception. Grounded in the hypothesis that targeted functional neurology treatments could significantly modulate these parameters, thereby enhancing physical performance and pain management, our research aimed to contribute novel insights into the efficacy of such interventions. The findings from our comprehensive assessments before and after the functional neurology intervention provide empirical evidence supporting the hypothesis. These results underscore the potential of functional neurology to induce meaningful improvements in neuromuscular function and psychophysiological health, highlighting its value as a therapeutic modality in both sports performance optimization and rehabilitation contexts.

The examination of algometry and hand strength variables post-functional neurology intervention revealed intriguing shifts, potentially enriching our comprehension of pain management and muscle strength rehabilitation strategies. Regarding the increase in PPT values, a previous study also found that interventions such as heavy rolling massage significantly increased it, suggesting a notable decrease in pain sensitivity [22]. This elevation in PPT aligns with the premise that mechanical stress or central nervous system modulation plays a critical role in reducing pain perception, a hypothesis supported by various mechanisms including the gate theory of pain and activation of mechanoreceptors and proprioceptors, which collectively could explain the observed improvements in our study. Conversely, examining the effects of dry needling, another intervention focusing on neuromuscular issues, showed increased corticospinal excitability and changes in mechanical pain sensitivity [23]. Other research has provided additional insights into the mechanisms and effects of neuromuscular interventions on pain and muscle strength. Ristorini et al. [24] demonstrated that dry needling significantly increased the pressure pain threshold in patients with myofascial pain syndrome, suggesting that such interventions can alter nociceptive processing pathways, potentially through mechanisms like those observed in our study. Similarly, Smith and Osborn [25] explored the psychological impact of chronic pain, highlighting the importance of considering both physiological and psychological factors in treatment approaches. Their findings suggest that interventions leading to pain reduction could also improve psychological well-being by alleviating the perceived assault on the self that chronic pain represents. This suggests that different therapeutic interventions can modulate pain perception and muscle function through diverse physiological pathways, providing a broadened perspective on the mechanisms underlying functional neurology’s impact. Specifically, the efficacy of functional neurology interventions in bolstering hand strength and reducing pain could be attributed to the tailored approach these treatments take towards rectifying dysfunctions within the nervous system, thereby facilitating improved neuromuscular responses [26].

The noted improvements in hand strength might be seen as complementary to the PPT findings, with both reflecting the neuroplastic potential of targeted interventions to enhance motor output and reduce pain. The enhancements observed in hand strength and PPT following functional neurology interventions reflect the neuroplastic capabilities of such treatments to improve motor output and mitigate pain. These findings align with recent research demonstrating the positive impact of neuromuscular exercises on pain reduction and the increase in active range of motion in conditions such as idiopathic frozen shoulder [27]. Additionally, the utility of neuromuscular exercise in reducing low back pain intensity and enhancing physical functioning among female healthcare workers was highlighted by other authors [28]. The enhancements in hand strength observed after functional neurology treatments can be contextualized within the framework of neuroplasticity, the brain’s ability to reorganize itself by forming new neural connections throughout life. This concept underpins the functional neurology approach, where treatments are tailored to stimulate the nervous system and improve its function through various methods, including physical exercises, balance and vestibular rehabilitation, and cognitive training. Such interventions aim to address dysfunctions in different parts of the nervous system, potentially leading to improved motor output and reduced pain by enhancing neuromuscular coordination and strength [29]. The targeted stimulation provided by these interventions not only fosters neural reorganization but also supports the strengthening of neural pathways critical for motor function and pain modulation. For instance, it was shown that balance and vestibular rehabilitation exercises can significantly influence the cerebellum’s capacity for coordination and balance, illustrating the practical application of neuroplastic principles in improving motor skills and reducing symptoms of dysfunction [30].

The observed improvements in hand strength following functional neurology treatments can indeed be contextualized within the broad scope of neuroplasticity, which is the foundation of the functional neurology approach. This concept highlights the brain’s remarkable capacity to adapt and rewire itself, a principle that is leveraged through various rehabilitative strategies to enhance both motor output and reduce pain. In line with this, the role of vestibular rehabilitation, a key component in the suite of functional neurology treatments, has been underscored by studies showing its efficacy in promoting central neuroplasticity. In this line, our findings highlight the role of the myotatic reflex in neuromuscular endurance under visual–vestibular stress. While previous studies have examined the impact of visual and vestibular stimuli on muscle function [6,11], this study specifically evaluates dysfunction in the myotatic reflex as a neuromuscular indicator.

The improvements in muscle endurance and pain perception following the functional neurology intervention suggest a potential role of sensorimotor recalibration and neuromuscular adaptation. Rather than attributing these changes broadly to neuroplasticity, they may be explained by enhanced proprioceptive integration and modulation of afferent sensory processing, which could contribute to improved neuromuscular efficiency [3]. The intervention’s targeted sensory stimulation may have enhanced the body’s ability to process visual, vestibular, and proprioceptive inputs, leading to functional improvements in motor control. However, since no direct neural imaging or electrophysiological assessment was conducted, conclusions about neuroplasticity remain speculative and require further investigation. For example, the significant increase in saccadic stimuli tolerance and hand strength observed in this study suggests that the intervention successfully activated neural pathways associated with motor control and endurance. Moreover, the reduction in pain perception, as indicated by higher pressure pain threshold values, may result from the modulation of nociceptive pathways through mechanisms such as the gate control theory of pain and the activation of descending inhibitory pathways. These processes highlight how targeted interventions can recalibrate neural networks to mitigate pain and improve functional outcomes. Additionally, the stabilization of critical flicker fusion threshold values points to the intervention’s potential in maintaining cortical arousal, ensuring optimal cognitive and physiological responses under stress. By leveraging the principles of neuroplasticity, functional neurology interventions create a holistic impact, addressing both localized neuromuscular dysfunctions and broader psychophysiological health, reinforcing their value as an innovative therapeutic modality. This form of therapy is designed to alleviate symptoms associated with vestibular disorders, including issues with balance and gait, by encouraging the brain to compensate for inner ear deficits through adaptation and habituation exercises [31]. This rehabilitative approach not only targets the visual system but also engages various neural circuits, contributing to the broader neuroplastic improvements observed in patients.

The improvements in visual function, as evidenced by increased tolerance to saccadic eye movements and enhanced CFFT values, can be attributed to the neuroplastic adaptations facilitated by functional neurology intervention. This form of therapy emphasizes the brain’s inherent ability to reorganize and form new neural connections in response to injury or dysfunction. For instance, the role of covert saccades, which are rapid eye movements that compensate for vestibular loss, underscores the adaptability of the visual system to maintain ocular and postural stability despite significant vestibular deficits [32]. In this line, the functional neurology intervention is shown to be more efficient than traditional interventions and highlights that the importance of establishing a home exercise program as part of vestibular rehabilitation treatment cannot be overstated [33], an approach that aligns with the broader understanding of neuroplasticity in rehabilitation, where targeted interventions and stimulation are essential for inducing the beneficial neural changes necessary for recovery from brain injuries [34], since in just one session, participants increased by 625% the number of saccadic stimuli they could tolerate without experiencing dysfunction. Since CFFT values remained stable in this study, the intervention did not significantly influence cortical arousal. This suggests that the observed improvements in neuromuscular endurance and pain perception were likely mediated by peripheral sensorimotor integration rather than central arousal mechanisms. While previous studies have linked CFFT increases to enhanced cognitive performance and vigilance [35], our findings indicate that the intervention primarily affected neuromuscular function rather than cortical arousal. The enhanced CFFT values could have direct implication in different areas such as sports performance and training [35,36], health [37], educational areas [38,39], as well as military environments [40,41], since in these situations a decrease in cortical arousal was found, and this intervention could improve the performance of participants as well as prevent for injuries.

The findings of this study highlight the potential effects of sensorimotor modulation in enhancing neuromuscular endurance and pain perception. While functional neurology interventions remain controversial due to a lack of extensive clinical validation, the results provide preliminary evidence supporting its utility in healthy individuals. Given that the intervention consisted of a single 20 min session, the observed improvements suggest that even short-duration neuromodulatory strategies may influence motor control mechanisms. However, further research is required to replicate these findings in different populations, particularly in clinical settings involving neurological disorders where sensorimotor dysfunctions are prevalent. In this line, while our findings indicate that functional neurology interventions can enhance neuromuscular performance and pain thresholds in healthy individuals, additional research is necessary to determine their efficacy in optimizing athletic performance and aiding in neurorehabilitation. Future studies should investigate these effects in competitive athletes and patients with neurodegenerative conditions to validate their broader therapeutic potential.

### 4.1. Practical Applications

The practical applications of this study extend into various domains, particularly in enhancing sports performance, rehabilitation protocols, and cognitive therapy. By demonstrating significant improvements in neuromuscular function and psychophysiological health through functional neurology interventions, our findings underscore the potential of targeted neurological treatments to optimize athletic performance. Athletes could benefit from personalized functional neurology programs designed to enhance proprioceptive feedback, balance, and visual–motor coordination, thereby reducing injury risk and improving competitive edge. In the realm of rehabilitation, the study’s insights into pain management and muscle strength recovery offer a novel approach for treating patients with chronic pain and neuromuscular disorders. Implementing functional neurology techniques could expedite recovery times, improve patient outcomes, and potentially reduce reliance on pharmacological interventions. Furthermore, the observed neuroplastic changes suggest that these interventions could be applied in cognitive therapy settings, aiding in the recovery of patients with neurodegenerative diseases or those recovering from neurological injuries. By harnessing the brain’s capacity for neuroplasticity, functional neurology could offer a complementary or alternative treatment pathway, enhancing quality of life and functional independence for a wide range of individuals.

### 4.2. Study Limitations and Future Research Lines

This study, while illuminating the benefits of functional neurology on saccadic stimuli dysfunction, presents several limitations that pave the way for future research endeavors. Firstly, the study’s participant pool, though adequate in size, was relatively homogenous in terms of health status and age. Expanding future studies to include a broader demographic, including individuals with specific neurological disorders or varying age groups, could elucidate the applicability of functional neurology across a wider spectrum of the population. Another limitation is the study’s design, which did not include a control group undergoing a placebo or alternative treatment. The inclusion of such a control would strengthen the causal inference between functional neurology interventions and observed improvements in neuromuscular function and psychophysiological health.

Moreover, the study primarily focused on short-term outcomes following functional neurology interventions. Longitudinal studies are needed to assess the sustainability of these benefits over time and to determine the optimal frequency and duration of treatment for long-lasting effects. Future research could also benefit from integrating more sophisticated neuroimaging techniques to visualize the neuroplastic changes purported to result from functional neurology interventions. Such insights would deepen our understanding of the neurological mechanisms underpinning the observed improvements, offering a more detailed map of how specific interventions impact neural pathways. Additionally, exploring the effects of functional neurology on a broader range of physiological and cognitive functions could unveil further applications of this approach. Studies examining its impact on mental health, cognitive decline, and neurodegenerative diseases could significantly expand the therapeutic scope of functional neurology. While the study aimed for homogeneity in participant health status and age, the reported age ranges (22 to 48 years in the experimental group, 20 to 35 years in the control group) provide a clearer picture of the sample’s distribution. Future research should further explore the effects of functional neurology interventions across a broader age spectrum to enhance generalizability. Lastly, comparative studies assessing the effectiveness of functional neurology against more traditional therapeutic modalities could highlight its relative strengths and limitations, guiding clinicians in selecting the most appropriate treatment strategies for their patients. By addressing these limitations and exploring proposed future research lines, the field can further validate and refine functional neurology as a potent tool for enhancing human health and performance.

## 5. Conclusions

This study provides compelling evidence that a single session of functional neurology intervention can significantly enhance neuromuscular endurance, improve pain thresholds, and modulate physiological responses associated with visual–motor integration in healthy participants. The findings suggest that targeted neuroplasticity-driven treatments may play a critical role in optimizing both performance and rehabilitation strategies. However, while these results highlight a promising therapeutic potential, the study’s exploratory nature and limited sample size necessitate further investigation to establish causality and generalizability. Future research should employ longitudinal designs and neurophysiological assessments to validate these effects and better understand the underlying neural mechanisms. These insights could contribute to refining neuromodulatory approaches in clinical and sports performance contexts, reinforcing the importance of integrating visual–motor interventions into broader neuromuscular rehabilitation frameworks.

## Figures and Tables

**Table 1:** Psychophysiological and physical performance measures in experimental and control groups in the different evaluation moments.

Group	Variable	1	2	3	4	F-Values of Wilks’ Lambda	sig	ηp2	Post Hoc Comparison
Experimental	PPT (kgf)	8.7 ± 5.1	8.5 ± 5.2	9.4 ± 5.1	10.2 ± 5.3	38.205	0.000	0.676	1 > 2 (0.000) 1 > 4 (0.000) 2 < 3 (0.011) 2 < 4 (0.000) 3 < 4 (0.000)
	Hand strength (N)	39.3 ± 11.3	39.8 ± 11.7	40.5 ± 11.4	40.5 ± 10.9	5.430	0.002	0.229	1 < 3 (0.006) 1 < 4 (0.035) 2 < 3 (0.041)
	CFFT (Hz)	35.2 ± 3.3	35.0 ± 2.7	34.7 ± 3.3	34.7 ± 2.7	4.303	0.008	0.190	2 > 4 (0.034)
	Blood oxygen saturation (%)	96.3 ± 2.0	96.8 ± 1.5	96.6 ± 1.7	97.1 ± 0.9	9.487	0.000	0.341	1 > 2 (0.000) 1 > 3 (0.008) 1 > 4 (0.000) 3 > 4 (0.002)
	Heart rate (bpm)	69.9 ± 9.7	67.2 ± 9.7	63.6 ± 7.8	65.4 ± 9.2	30.416	0.000	0.624	1 > 2 (0.000) 1 > 3 (0.000) 1 > 4 (0.000) 2 > 3 (0.032)
Control	PPT (kgf)	7.4 ± 2.5	7.2 ± 2.0	7.1 ± 1.8	7.2 ± 2.3 * (0.034)	1.150	0.361	0.187	
	Hand strength (N)	42.0 ± 10.2	42.0 ± 9.8	41.8 ± 9.5	40.9 ± 8.9	1.019	0.383	0.113	
	CFFT (Hz)	36.4 ± 3.5	35.3 ± 2.1	35.3 ± 2.2	35.4 ± 2.2	0.894	0.467	0.152	
	Blood oxygen saturation (%)	96.5 ± 1.3	96.8 ± 0.8	96.6 ± 0.9	96.3 ± 0.7	4.718	0.016	0.485	
	Heart rate (bpm)	82.3 ± 15.5	80.9 ± 13.2	78.5 ± 15.2	79.3 ± 14.3	2.481	0.070	0.115	

Baseline measurement (1); post-indicator muscle failure (pre-intervention) (2); post-functional neurology treatment (3); post-indicator muscle failure (post-intervention) (4); pressure pain threshold (PPT), critical flicker fusion threshold (CFFT). Results are presented as means ± standard deviations. GLM (General Linear Model) results are shown for within-group repeated measures comparisons across evaluation moments. Independent *t*-test results were applied for between-group comparisons at moment 4; the asterisk (*) in the control group for PPT at moment 4 indicates a *p*-value of 0.034 in the GLM analysis, although post hoc comparisons did not reveal statistically significant differences; the *p*-value of 0.016 in blood oxygen saturation in the control group reflects a statistically significant result from GLM analysis; however, changes were small and not physiologically meaningful.

## Data Availability

The data that support the findings of this study are available from the corresponding authors upon reasonable request. The data are not publicly available due to restrictions related to participant confidentiality.
